# Inhibition of the aberrant A1CF-FAM224A-miR-590-3p-ZNF143 positive feedback loop attenuated malignant biological behaviors of glioma cells

**DOI:** 10.1186/s13046-019-1200-5

**Published:** 2019-06-11

**Authors:** Yichen Song, Lianqi Shao, Yixue Xue, Xuelei Ruan, Xiaobai Liu, Chunqing Yang, Jian Zheng, Shuyuan Shen, Jiajia Chen, Zhen Li, Yunhui Liu

**Affiliations:** 1 Department of Neurosurgery, Shengjing Hospital of China Medical University, Shenyang, 110004 China; 2 Liaoning Clinical Medical Research Center in Nervous System Disease, Shenyang, 110004 China; 3 Key Laboratory of Neuro-oncology in Liaoning Province, Shenyang, 110004 China; 4 Department of Neurobiology, School of Life Sciences, China Medical University, Shenyang, 110122 China; 5 Key Laboratory of Cell Biology, Ministry of Public Health of China, China Medical University, Shenyang, 110122 China; 6 Key Laboratory of Medical Cell Biology, Ministry of Education of China, China Medical University, Shenyang, 110122 China

**Keywords:** Glioma, LncRNAs, A1CF, FAM224A, miR-590-3p, ZNF143, ASAP3

## Abstract

**Background:**

Glioma is the most common and lethal type of malignant brain tumor. Accumulating evidence has highlighted that RNA binding protein APOBEC1 complementation factor (A1CF) is involved in various cellular processes by modulating RNA expression, and acts as an oncogene in breast cancer. However, the function of A1CF in glioma remained unclear.

**Methods:**

Quantitative RT-PCR and western blot analysis were employed to detect the expression levels of A1CF, lncRNA family with sequence similarity 224 member A (FAM224A), miR-590-3p, zinc finger protein 143 (ZNF143) and ArfGAP with SH3 domain, ankyrin repeat and PH domain 3 (ASAP3) in glioma tissues and cell lines. The Cell Counting Kit-8 assay, migration and invasion assays, and flow cytometry analysis were conducted to evaluate the function of A1CF, FAM224A, miR-590-3p, ZNF143 and ASAP3 in the malignant biological behaviors of glioma cells. Moreover, luciferase reporter, RIP and ChIP assays were used to investigate the interactions among A1CF, FAM224A, miR-590-3p, ZNF143, ASAP3 and MYB. Finally, the xenograft tumor growth assay further ascertained the biological roles of A1CF, FAM224A and miR-590-3p in glioma cells.

**Results:**

A1CF was upregulated and functioned as an oncogene via stabilizing and increasing FAM224A expression; moreover, high A1CF and FAM224A expression levels indicated a poorer prognosis for glioma patients. Conversely, miR-590-3p was downregulated and exerted a tumor-suppressive function in glioma cells. Inhibition of A1CF significantly restrained cell proliferation, migration and invasion, and promoted apoptosis by upregulating miR-590-3p in a FAM224A-dependent manner. FAM224A was a molecular sponge of miR-590-3p and they were in an RNA-induced silencing complex. ZNF143 was upregulated in glioma tissues and cell lines. MiR-590-3p could negatively modulate the expression of ZNF143 via binding to the ZNF143 3′ UTR. Moreover, ZNF143 participated in miR-590-3p-induced tumor-suppressive activity on glioma cells. ASAP3 and MYB were transcriptionally activated by ZNF143, and importantly, ZNF143 could directly target the promoter of FAM224A and stimulate its expression, collectively forming a positive feedback loop.

**Conclusions:**

The present study clarifies that the A1CF-FAM224A-miR-590-3p-ZNF143 positive feedback loop conducts critical regulatory effects on the malignant progression of glioma cells, which provides a novel molecular target for glioma therapy.

**Electronic supplementary material:**

The online version of this article (10.1186/s13046-019-1200-5) contains supplementary material, which is available to authorized users.

## Background

Glioma is considered to be the most common and lethal type of malignant brain tumor [1]. Despite recent advances in surgery, chemotherapy and radiotherapy, the prognosis of glioma has not improved significantly, with patients having a 5-year survival rate of only 3.3% [2, 3]. Therefore, precise gene-targeted therapy is expected to become an effective therapeutic strategy for glioma.

RNA binding proteins (RBPs) play a pivotal role in the post-transcriptional regulation of gene expression and exert important functions in various biological processes [4–6]. APOBEC1 complementation factor (A1CF) participates in the post-transcriptional cytidine (C) to uridine (U) RNA editing of apolipoprotein B (APOB) mRNA by cooperating with its partner APOBEC1, further modulating lipid metabolism [7]. Recent studies have indicated that A1CF is closely related to malignant tumor progression. For example, A1CF promotes the proliferation of breast cancer cells via upregulating interleukin-6 expression [8]. However, the potential role of A1CF in glioma remains unclear.

Long non-coding RNAs (lncRNAs) are a type of non-coding RNAs with transcripts longer than 200 nucleotides in length with limited protein-coding ability. Evidence suggests that lncRNAs act as key modulators in the occurrence and development of human tumors. For example, dysregulation of TUG1, HOTAIR and MALAT1 contributes to the malignant progression of esophageal squamous cell carcinoma, breast cancer and pancreatic cancer [9–11]. Moreover, overexpression of HCP5 and Linc00152 facilitates the malignant biological behaviors of glioma cells [12, 13]. However, to date, there have been no reports on the function of the lncRNA family with sequence similarity 224 member A (FAM224A) in human glioma.

MicroRNAs (miRNAs) are a class of endogenous small non-coding RNAs of approximately 20–22 bp in length. They are involved in the regulation of post-transcriptional gene expression mainly through binding to the 3′ UTR of target genes and causing either degradation or repression of mRNAs [14, 15]. MiR-590-3p serves as a critical modulator on tumorigenesis and malignant progression. It exerts tumor-suppressive functions in hepatocellular carcinoma as well as head and neck squamous cell carcinoma [16, 17]. In particular, miR-590-3p has also been found to be downregulated in glioma and impedes the malignant development of glioma cells through targeting ZEB1 and ZEB2 [18]. However, the detailed regulatory mechanism of miR-590-3p in glioma still remains unclarified.

Transcription factor zinc finger protein 143 (ZNF143) is a human homolog of the *Xenopus* transcriptional activator Staf and its expression can be induced by treatment with various DNA-damaging agents [19]. Multiple reports have proven that ZNF143 plays a role in cancer drug resistance and the DNA repair of tumor cells [20, 21]. Recently, ZNF143 was reported to be upregulated in lung adenocarcinoma, and was a predictor of high proliferating activity and poor prognosis in patients with lung adenocarcinoma [22]. To date, the mechanisms underlying the function of ZNF143 in glioma have not been elucidated. ArfGAP with SH3 domain, ankyrin repeat and PH domain 3 (ASAP3), an important member of the ArfGAP family, plays a carcinogenic role in various tumors. For example, the expression of ASAP3 is elevated in non-small cell lung cancer, hepatocellular carcinomas and colorectal cancer, which contributes to malignant progression and indicates poor survival outcomes in patients [23–25]. However, the biological function of ASAP3 in glioma is still undefined.

In the present study, the endogenous expression of A1CF, FAM224A, miR-590-3p and ZNF143 were investigated in glioma tissues and cell lines. Their roles in modulating malignant progression of glioma and the cross-talk between A1CF, FAM224A, miR-590-3p and ZNF143 were elucidated. Our findings may provide a novel target for glioma therapy.

## Methods

### Clinical specimens

A total of 43 glioma specimens and 6 normal brain tissues (NBTs) were obtained from the Department of Neurosurgery, Shengjing Hospital of China Medical University. NBTs were the rejected materials obtained from surgeries of severe intracerebral hemorrhage, brain trauma and epilepsy. After surgical resection, all human tissues were immediately frozen in liquid nitrogen for long-term preservation. According to the WHO classification of CNS tumors, glioma tissues were divided into four grades: 1. Grade I (WHO I, *n* = 8); 2. Grade II (WHO II, *n* = 10); 3. Grade III (WHO III, *n* = 11); 4. Grade IV (WHO IV, *n* = 14). This research had obtained approval of Shengjing Hospital Ethical Committee.

### Cell lines and cultures

Normal human astrocyte (NHA) cells were purchased from Sciencell Research Laboratories (Carlsbad, CA, USA) and human glioma U87, U251 and HEK293T cells were purchased from Shanghai Institutes for Biological Sciences Cell Resource Center. NHA cells grown in astrocyte medium and U87, U251, HEK293T cells were cultured in DMEM/high glucose mixed with 10% FBS (Gibco, Carlsbad, CA, USA). All cells were maintained in a humidified incubator at 37 °C with 5% CO2.

### Cell transfection

The short-hairpin RNA (shRNA) against A1CF, ZNF143 and ASAP3 genes (A1CF (−), ZNF143 (−) and ASAP3 (−)), as well as their negative control (A1CF (−)-NC, ZNF143 (−)-NC and ASAP3 (−)-NC) were designed and cloned in pGPU6/GFP/Neo vector by GenePharama (Shanghai, China). The shRNA against FAM224A gene (FAM224A (−)) and its negative control (FAM224A (−)-NC) were designed and cloned in U6-MCS-Ubiquitin-Cherry-IRES-puromycin vector by GENECHEM (Shanghai, China). Full-length A1CF, FAM224A, ZNF143 and ASAP3 genes (A1CF (+), FAM224 (+), ZNF143 (+) and ASAP3 (+)) and their negative control (A1CF (+)-NC, FAM224 (+)-NC, ZNF143 (+)-NC and ASAP3 (+)-NC) were ligated into pIRES2-EGFP (GenScript, Piscataway, NJ, USA). MiR-590-3p agomir (pre-miR-590-3p, sequence: 5′- UAAUUUUAUGUAUAAGCUAGUUAGCUUAUACAUAAAAUUAUU-3′) and miR-590-3p antagomir (anti-miR-590-3p, sequence: 5′-ACUAGCUUAUACAUAAAAUUA-3′) and their negative control (pre-NC and anti-NC) were synthesized by GenePharma. Lipofectamine 3000 and Opti-MEM I (Life Technologies, Waltham, MA) were used to transfect cells with the plasmids above for overexpression and knockdown of corresponding genes when cells reaching 70–80% confluence in a 24-well plate. Then stable transfected cells were selected by G418 or puromycin (Sigma-Aldrich, St Louis, MO, USA) screening. The transfection efficacy was detected by qRT-PCR or western blot (Additional file 5: Figure S5). To determine the effects of A1CF on glioma, cells were divided into five groups: Control, A1CF (+)-NC, A1CF (+), A1CF (−)-NC and A1CF (−). To evaluate the effects of FAM224A on glioma, cells were divided into five groups: Control, FAM224A (+)-NC, FAM224A (+), FAM224A (−)-NC and FAM224A (−).To investigate the effects of both knockdown of A1CF and FAM224A on glioma, cells were divided into five groups: Control, A1CF (−)-NC + FAM224A (−)-NC, A1CF (−) + FAM224A (−)-NC, A1CF (−)-NC + FAM224A (−) and A1CF (−) + FAM224A (−). To determine the effects of miR-590-3p on glioma, cells were divided into five groups: Control, pre-NC, pre-miR-590-3p, anti-NC and anti-miR-590-3p. To evaluate whether tumor-suppressive effects of FAM224A knockdown were mediated by miR-590-3p, cells were divided into five groups: Control, FAM224A (−)-NC + pre-NC, FAM224A (−) + pre-miR-590-3p, FAM224A (−)-NC + anti-NC and FAM224A (−) + anti-miR-590-3p. To investigate the effects of ZNF143 on glioma, cells were divided into five groups: Control, ZNF143 (+)-NC, ZNF143 (+), ZNF143 (−)-NC and ZNF143 (−). To determine whether ZNF143 is involved in the miR-590-3p-induced effects on the behaviors of glioma, cells were divided into five groups: Control, pre-NC + ZNF143 (+)-NC, pre-miR-590-3p + ZNF143 (+)-NC, pre-NC + ZNF143 (+) and pre-miR-590-3p + ZNF143 (+). To evaluate the effects of ASAP3 on glioma, cells were divided into five groups: Control, ASAP3 (+)-NC, ASAP3 (+), ASAP3 (−)-NC and ASAP3 (−).

### RNA isolation and quantitative RT-PCR (qRT-PCR)

Total RNA was extracted from the tissues and cells according to Trizol reagent (Life Technologies Corporation, Carlsbad, CA, USA) manufacturer’s instructions. SYBR Prime-Script RT-PCR Kit (TakaraBio, Japan) was applied to examine the mRNA expression levels of A1CF, FAM224A, ZNF143, ASAP3, MYB and GAPDH. TaqMan MicroRNA Reverse Transcription kit and TaqMan Universal Master Mix II (Applied Biosystems, Foster City, CA, USA) was employed to evaluate the expression levels of miR-590-3p and U6. After normalizing to the endogenous control GAPDH or U6, the relative expression values were calculated to represent fold change in gene expression by relative quantification (2^−ΔΔCt^ method).

### LncRNAs microarray analysis

The sample preparation, microarray hybridization and lncRNAs analysis was performed by Kangchen Bio-tech (Shanghai, China).

### Western blot analysis

Equal amounts of protein samples were electrophoresed in 8% sodium dodecyl sulfate polyacrylamide gel electropheresis (SDS-PAGE) and then transferred to PVDF membranes (Millipore, Shanghai, China). After blocking in 5% non-fat milk for 2 h at room temperature, the membranes went immunoblotting against A1CF (1:1000, SAB, No: 39439, USA), ZNF143 (1:1000, Proteintech, No: 16618–1-AP, USA), ASAP3 (1:200, Santa Cruz Biotechnology, No: sc-135,740, USA), MYB (1:1000, SAB, No: 37435, USA) and GAPDH (1:5000, Proteintech, No: 10494–1-AP, USA). After incubated 2 h with HRP-conjugated secondary antibodies at room temperature, protein bands were visualized by ECL (Beyotime) and measured by ECL Detection Systems (Thermo Scientific, Beijing, China). The relative expression level was calculated based on the internal control GAPDH.

### Cell proliferation assay

U87 and U251 cells were seeded in 96-well plate (2000 cells per well) and cultured at 37 °C for 24 h. Then 10 μl of Cell Counting Kit-8 (CCK-8, Dojin, Japan) solution was added into each well and incubated at 37 °C for another 2 h. The absorbance was measured and recorded at 450 nm.

### Cell migration and invasion assays

For migration assay, U87 and U251 cells were resuspended in serum-free medium at a density of 2 × 10^5^ cells/ml, then 100 μl cell suspension was seeded into the upper chamber of a 24-well transwell chamber (Costar, Corning, NY, USA), meanwhile 600 μl DMEM/high glucose medium contained 10% FBS was added to the lower chamber. After incubation at 37 °C for 24 h, cells were fixed with methanol and stained with 20% Giemsa solution for 30 min at 37 °C. Then cell numbers were counted by average of five random fields under an inverted microscope. For invasion assay, 80 μl of 50 ng/μl Matrigel solution (BD, Franklin Lakes, NJ, USA) was pre-coated on the transwell membrane.

### Apoptosis analysis

Annexin V-PE/7-AAD (BD, Biosciences) was employed to detect cell apoptosis. According to the manufacturer’s instruction, U87 and U251 cells were collected and washed with cold phosphate-buffered saline twice, then stained 15 min with Annexin V-PE/7AAD at room temperature. The apoptotic fractions were then analyzed by flow cytometry (FACScan, BD Biosciences).

### Dual-luciferase reporter assay

The predicted miR-590-3p binding sequence in FAM224A and ZNF143 3′ UTR sequence and their corresponding mutant sequence were cloned into pmirGLO Dual-Luciferase Vector to construct luciferase reporter vector (FAM224A-Wt or -Mut and ZNF143–3′ UTR-Wt or -Mut; GenePharma). HEK293T cells were seeded into a 96-well plate and co-transfected with the indicated vectors and miR-590-3p agomir or its negative control, respectively. Dual-luciferase assay was performed 48 h after transfection, the relative luciferase activity was normalized to renilla luciferase activity and calculated by Dual Luciferase Reporter Assay System (Promega, Madison, WI, USA).

### RNA immunoprecipitation (RIP)

EZ-Magna RIP kit (Millipore, Billerica, MA) was applied to conduct RIP assay according to the manufacturer’s protocol. Whole cell lysate were incubated with RIP magnetic beads conjugated with anti-Argonaute2 antibody and normal mouse IgG (Millipore). After incubating with Proteinase K buffer, immunoprecipitated RNA was obtained. Finally, the presence of the binding targets was validated by qRT-PCR.

### Nascent RNA capture assay

Click-iT Nascent RNA Capture Kit (Life Technologies Corporation, Eugene, Oregon, USA) was used to detect the relative expression of nascent FAM224A according to the manufacturer’s instruction. In brief, total RNA was obtained after the nascent RNA was labeled with 0.2 mM EU and treated for 4 h. Add UltraPureTM glycogen, ammonium acetate and 75% ethanol into the preparation of Click-iT reaction cocktail and centrifugal RNA. Formulated reaction mixture with binding buffer, RNA Recombinant Ribonuclease inhibitor and DNase/RNase-free water. Then fix the beads with DynaMag-2 magnet. In order to release cDNA from the magnetic beads, heating the magnetic beads suspension to 70 °C with adding SuperScipt Enzyme Mix and intermittently vortexed oscillation. Finally, the relative expression of nascent RNA in the supernatant was examined by qRT-PCR.

### RNA stability measurement

Total RNA of HEK293 cells transfected with sh-A1CF and its negative control was harvested at the indicated time points after treatment with Actinomycin D (5 mg/ml, Sigma-Aldrich, St Louis, MO, USA) or DMSO as a control. Then the remaining levels were analyzed by qRT-PCR.

### Chromatin immunoprecipitation assay (ChIP)

Simple ChIP Enzymatic Chromatin IP Kit (Cell Signaling Technology) was uilized for ChIP assay based on the manufacturer’s protocol. Briefly, cells were crosslinked with EBM-2 containing 1% formaldehyde for 10 min and collected in lysis buffer. Then chromatin was digested by micrococcal nuclease. The immunoprecipitation sample was incubated with 3 μg of anti-ZNF143 antibody or normal rabbit IgG, then gently rotated overnight at 4 °C after treating with Protein G Agarose Beads. DNA crosslinks were reversed by 5 M NaCl and proteinase K, finally were purified. Immunoprecipitated DNA was amplified by PCR with their specific primers.

### Tumor xenografts in nude mice

Stable expression glioma cells for in vivo study were established as following steps. Lentivirus encoding miR-590-3p was generated using pLenti6.3/V5eDEST Gateway Vector Kit (Life Technologies Corporation), while shRNA targeting human A1CF and FAM224A were ligated into the LV3-CMV-GFP-Puro vector (GenePharma). After infection, the stable expressing cells of A1CF (−), FAM224A (−) and pre-miR-590-3p were established. Subsequently, the miR-590-3p lentivirus were transduced in stable expressing cells of A1CF (−) and FAM224A (−) to produce A1CF (−) + FAM224A (−) + pre-miR-590-3p cells. All research methods were conducted strictly in accordance with the protocol of Care and Use of Laboratory Animals, moreover, approvals from the Administrative Panel on Laboratory Animal Care of the Shengjing Hospital also were achieved. Four-week-old BALB/C athymic nude mice were purchased from the National Laboratory Animal Center (Beijing, China), and divided into five groups (randomized to each group by two performers in a blinded manner, *n* = 10 per group): Control, A1CF (−), FAM224A (−), pre-miR-590-3p and A1CF (−) + FAM224A (−) + pre-miR-590-3p. Each mice was subcutaneously injected 3 × 10^5^ cells in the right flanks for subcutaneous implantation assay. Tumor volume was measured every 4 days using the formula: volume (mm^3^) = length × width^2^/2. Forty-four days after injection, the tumor-bearing mice were executed and tumors were isolated. The orthotopic inoculation experiments were conducted by stereotactically implanting 3 × 10^5^ cells into the mice right striatum. Numbers of surviving or dead nude mice were recorded at any time, and survival analysis was performed using Kaplan–Meier survival curve.

### Statistical analysis

Experimental data are presented as the mean ± standard deviation (SD) from at least five independent experiments. SPSS 22.0 software was used for statistical analysis with the Student’s *t*-test, one-way ANOVA, Pearson chi-square test or Log-rank test. Differences were considered statistically significant when *P* < 0.05.

## Results

### A1CF exerts a carcinogenic role in glioma cells via stabilizing and upregulating FAM224A

**Fig. 1:**
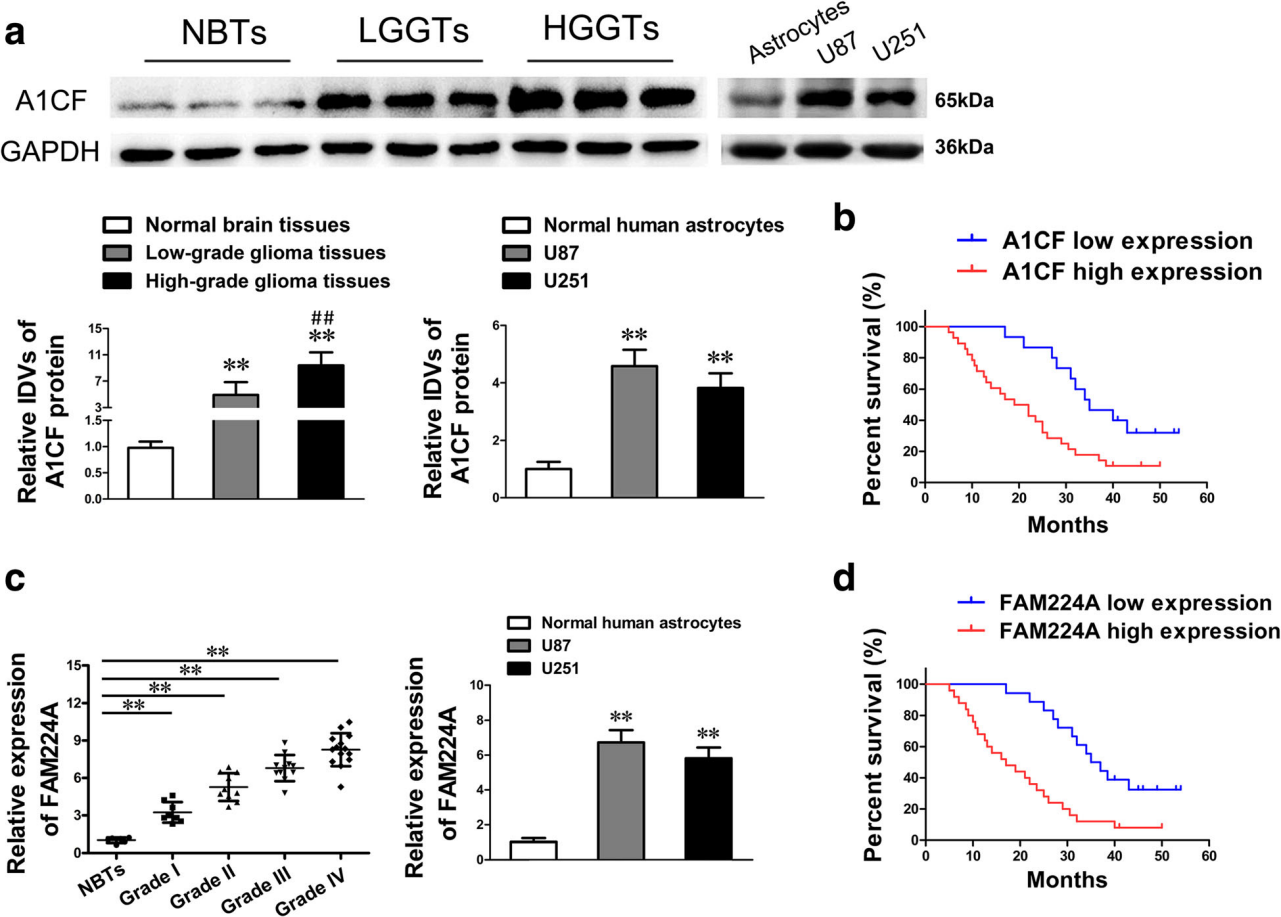
A1CF and FAM224A played an oncogenic role in glioma. **a**. The expression levels of A1CF were upregulated in glioma tissues (left) and cells (right). Left: Data are presented as the mean ± SD (*n* = 6, NBTs; *n* = 18, Low-grade glioma tissues, including Grade I and Grade II; *n* = 25, High-grade glioma tissues, including Grade III and Grade IV). ^**^P < 0.01 vs. Normal brain tissues. ^##^*P* < 0.01 vs. Low-grade glioma tissues. Right: Data are presented as the mean ± SD (*n* = 5, each group). ^**^P < 0.01 vs. NHA. **b**. Kaplan-Meier survival analyses showed that the glioma patients with high expression of A1CF indicated poorer overall survival (log-rank test, *P* = 0.0046). The mean of A1CF expression was used as cut-off. **c.** The expression levels of FAM224A were upregulated in glioma tissues of different grades (left) and cells (right). Left: Data are presented as the mean ± SD (*n* = 6, NBTs; *n* = 8, Grade I; *n* = 10, Grade II; *n* = 11, Grade III; *n* = 14, Grade IV). ^**^P < 0.01 vs. NBTs. Right: Data are presented as the mean ± SD (*n* = 5, each group). ^**^P < 0.01 vs. NHA. **d**. Glioma patients with high expression of FAM224A exhibited worse overall survival (log-rank test, *P* = 0.0004)

The expression levels of A1CF in NBTs, glioma tissues, NHA and glioma cell lines were detected by western blot analysis. The results showed that A1CF was significantly upregulated in glioma tissues and cell lines when compared with NBTs and NHA. The expression level of A1CF was positively correlated with the pathological grade of glioma (Fig. 1a). Moreover, Kaplan-Meier survival analyses and the log-rank test in 43 glioma patients suggested that higher A1CF expression indicated worse overall survival (Fig. 1b). The correlation analyses between A1CF expression levels and the clinicopathological features of 43 glioma patients are also displayed in (Additional file 6: Table S1). LncRNAs microarray analysis revealed that FAM224A was remarkably downregulated in glioma cells following knockdown of A1CF, implying that FAM224A may participate in A1CF-induced modulation on glioma cells (Additional file 1: Figure S1). Further, qRT-PCR showed that FAM224A was significantly upregulated in glioma tissues and cell lines when compared with NBTs and NHA (Fig. 1c). Furthermore, Kaplan-Meier survival analyses demonstrated that higher FAM224A expression levels led to poorer prognosis of glioma patients and were associated with advanced pathological grade tumors in 43 glioma patients (Fig. 1d and Additional file 6: Table S2). Subsequently, the functions of A1CF and FAM224A in glioma cells were examined by CCK-8 assay, flow cytometry analysis, and migration and invasion assays. As shown in Additional file 2: Figure S2 a–f, knockdown of A1CF or FAM224A obviously inhibited cell proliferation, migration and invasion, while promoted apoptosis in glioma cells when compared with the negative control groups. These results suggested that both A1CF and FAM224A played oncogenic roles in glioma cells.

**Fig. 2:**
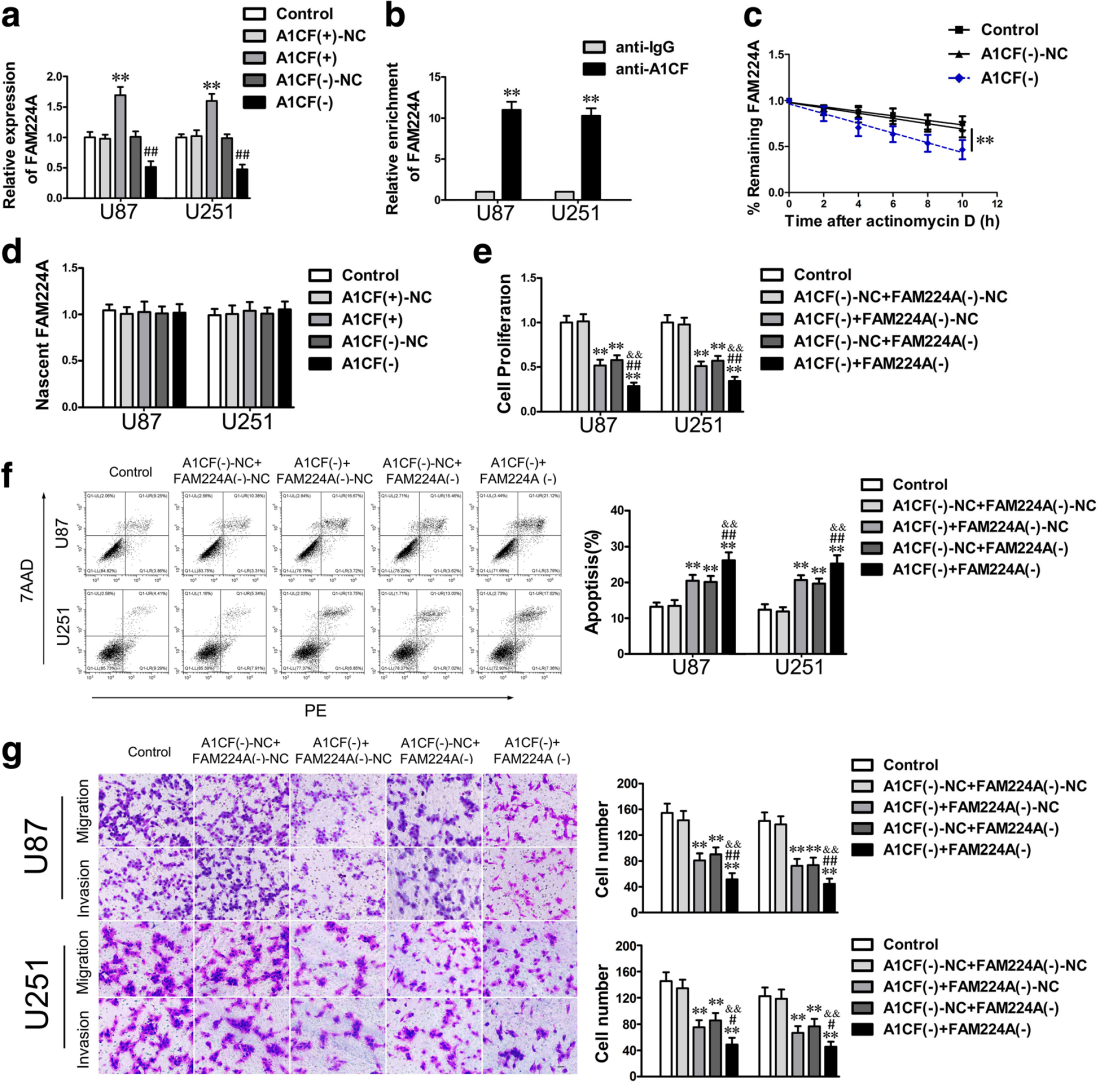
A1CF bound to FAM224A and strengthened its stability. **a**. The FAM224A expression of glioma cells treated with altering A1CF expression was showed. Data represent mean ± SD (*n* = 5, each group). ^**^P < 0.01 vs. A1CF (+)-NC group (negative control); ^##^*P* < 0.01 vs. A1CF (−)-NC group (negative control). **b.** FAM224A was confirmed in the A1CF complex. Data represent mean ± SD (*n* = 5, each group). ^**^P < 0.01 vs. anti-IgG group. **c.** The remaining levels of FAM224A at the different time points treated with actinomycin D were indicated. **d.** Nascent RNA capture assay was used to evaluate the nascent FAM224A in A1CF (+) and A1CF (−) group. **e-g.** CCK-8 assay, flow cytometry analysis and migration and invasion assays were used to detected the impacts of A1CF and FAM224A knockdown on biological behaviors of glioma cells. Data are presented as the mean ± SD (*n* = 5, each group). ^**^P < 0.01 vs. A1CF (−)-NC + FAM224 (−)-NC; ^##^*P* < 0.01 vs. A1CF (−) + FAM224 (−)-NC; ^#^*P* < 0.05 vs. A1CF (−) + FAM224 (−)-NC; ^&&^*P* < 0.01 vs. A1CF (−)-NC + FAM224 (−) group. Scale bar of migration and invasion assays represent 40 μm

Next, we investigated the correlation between A1CF and FAM224A. Using a bioinformatics database (Starbase), we predicted that A1CF might bind to FAM224A. As shown in Fig. 2a, knockdown of A1CF dramatically decreased FAM224A expression. In addition, an RIP assay indicated that enrichment of FAM224A was higher in the anti-A1CF group than in the anti-IgG group (Fig. 2b). Subsequently, RNA stability and nascent RNA capture assays were utilized to further clarify the mechanism of regulation in FAM224A expression by A1CF. The results showed that A1CF knockdown markedly reduced the half-life of FAM224A, whereas the nascent FAM224A was not affected (Fig. 2c and d). Finally, the malignant biological behaviors were evaluated in glioma cells co-transfected with sh-A1CF and sh-FAM224A. The results showed that simultaneously knockdown of A1CF and FAM224A obviously restrained the biological behaviors of glioma cells when compared with the negative control groups (Fig. 2e−g). These results indicated that A1CF exerted carcinogenic functions in glioma cells via stabilizing and upregulating FAM224A expression.

### MiR-590-3p is downregulated and manifests a tumor-suppressor in glioma cells

**Fig. 3:**
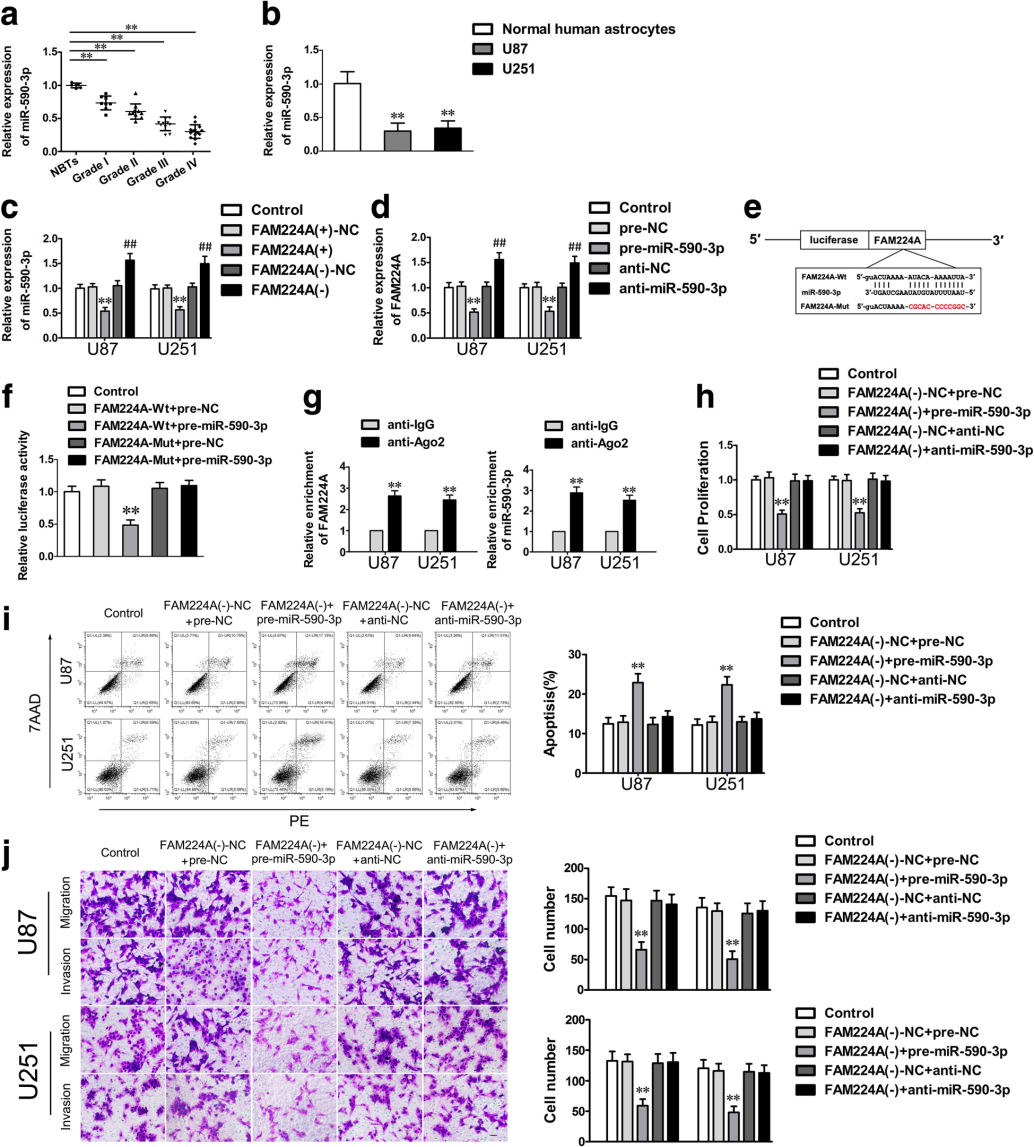
MiR-590-3p could target FAM224A and mediate the inhibitory effects of FAM224A knockdown on glioma cells. **a**. The expression levels of miR-590-3p were downregulated in glioma tissues. Data are presented as the mean ± SD (*n* = 6, NBTs; *n* = 8, Grade I; *n* = 10, Grade II; *n* = 11, Grade III; *n* = 14, Grade IV). ^**^P < 0.01 vs. NBTs. **b.** The expression levels of miR-590-3p in NHA, U87 and U251 cells were lowexpressed. Data are presented as the mean ± SD (*n* = 5, each group). ^**^P < 0.01 vs. NHA group. **c.** The miR-590-3p expression of glioma cells treated with altering FAM224A expression was exhibited. **d.** The FAM224A expression of glioma cells transfected with miR-590-3p agomir or antagomir was showed. **e.** The potential miR-590-3p binding sequence and the designed mutant sequence of FAM224A were indicated. **f.** Relative luciferase activity was detected after cells were co-transfected with pre-miR-590-3p and FAM224A-Wt or FAM224A-Mut. Data were given as mean ± SD (*n* = 5, each group). ^**^P < 0.01 vs. FAM224A-Wt + pre-NC group. **g.** RIP assay validated that FAM224A and miR-590-3p were involved in an RISC. Data represent mean ± SD (*n* = 5, each group). ^**^P < 0.01 vs. anti-IgG group. **h-j.** CCK-8 assay, flow cytometry analysis and migration and invasion assays were employed to investigate the biological behaviors of glioma cells co-transfected with sh-FAM224A and miR-590-3p agomir or antagomir. Data are presented as the mean ± SD (*n* = 5, each group). ^**^P < 0.01 vs. FAM224A (−)-NC + pre-NC group. Scale bar of migration and invasion assays represent 40 μm

The miR-590-3p expression levels, as shown in Fig. 3a and b, were proven to be significantly downregulated in glioma tissues and in U87 and U251 cell lines, compared with NBTs and NHA, respectively. Similarly, the expression level of miR-590-3p was negatively correlated with the progression of glioma pathological grade (Fig. 3a). To investigate the functions of miR-590-3p in glioma, we assessed the malignant biological behaviors of U87 and U251 cells treated with altered miR-590-3p expression levels. The results showed that overexpression of miR-590-3p significantly decreased the proliferation ability while facilitated apoptosis of glioma cells compared with the pre-NC group (Additional file 3: Figure S3a and b). Moreover, migration and invasion assays revealed an obvious decline in cell numbers when miR-590-3p was restored (Additional file 3: Figure S3c). The evidence above inferred that miR-590-3p conducted tumor inhibitory effects on glioma cells.

### FAM224A targets miR-590-3p and negatively regulates its expression

Using a bioinformatics database (Starbase), we found that FAM224A was a putative target of miR-590-3p. Further, we showed that the expression of miR-590-3p was significantly upregulated in glioma cells following FAM224A knockdown (Fig. 3c). Conversely, FAM224A expression was remarkably increased in glioma cells when miR-590-3p was downregulated (Fig. 3d). As shown in Fig. 3e and f, a dual-luciferase assay further elucidated the potential binding site and the molecular mechanism responsible for the interaction between FAM224A and miR-590-3p. Moreover, the results of an RIP assay showed that the enrichment of both FAM224A and miR-590-3p was increased in the anti-Ago2 group versus the anti-IgG group, suggesting that FAM224A and miR-590-3p were involved in an RNA-induced silencing complex (Fig. 3g). As suggested above, these results revealed that FAM224A directly targeted miR-590-3p and that together they formed a reciprocal repression feedback loop.

### Silencing miR-590-3p mediates the tumor-suppressive effects of FAM224A knockdown on glioma cells

To explore whether miR-590-3p influences the inhibitory functions of FAM224A knockdown, U87 and U251 cells were co-transfected with sh-FAM224A and pre- or anti-miR-590-3p. Subsequent analysis of biological behaviors suggested that FAM224A knockdown, which was associated with the overexpression of miR-590-3p, remarkably attenuated proliferation, migration and invasion, but promoted cell apoptosis when compared with the FAM224A (−)-NC + pre-NC group (Fig. 3h–j). Furthermore, reduction of miR-590-3p obviously rescued the repression of malignant progression of glioma cells induced by FAM224A downregulation (Fig. 3h–j).

### Knockdown of ZNF143 restrained cell proliferation, migration and invasion of glioma cells while promoting apoptosis

**Fig. 4:**
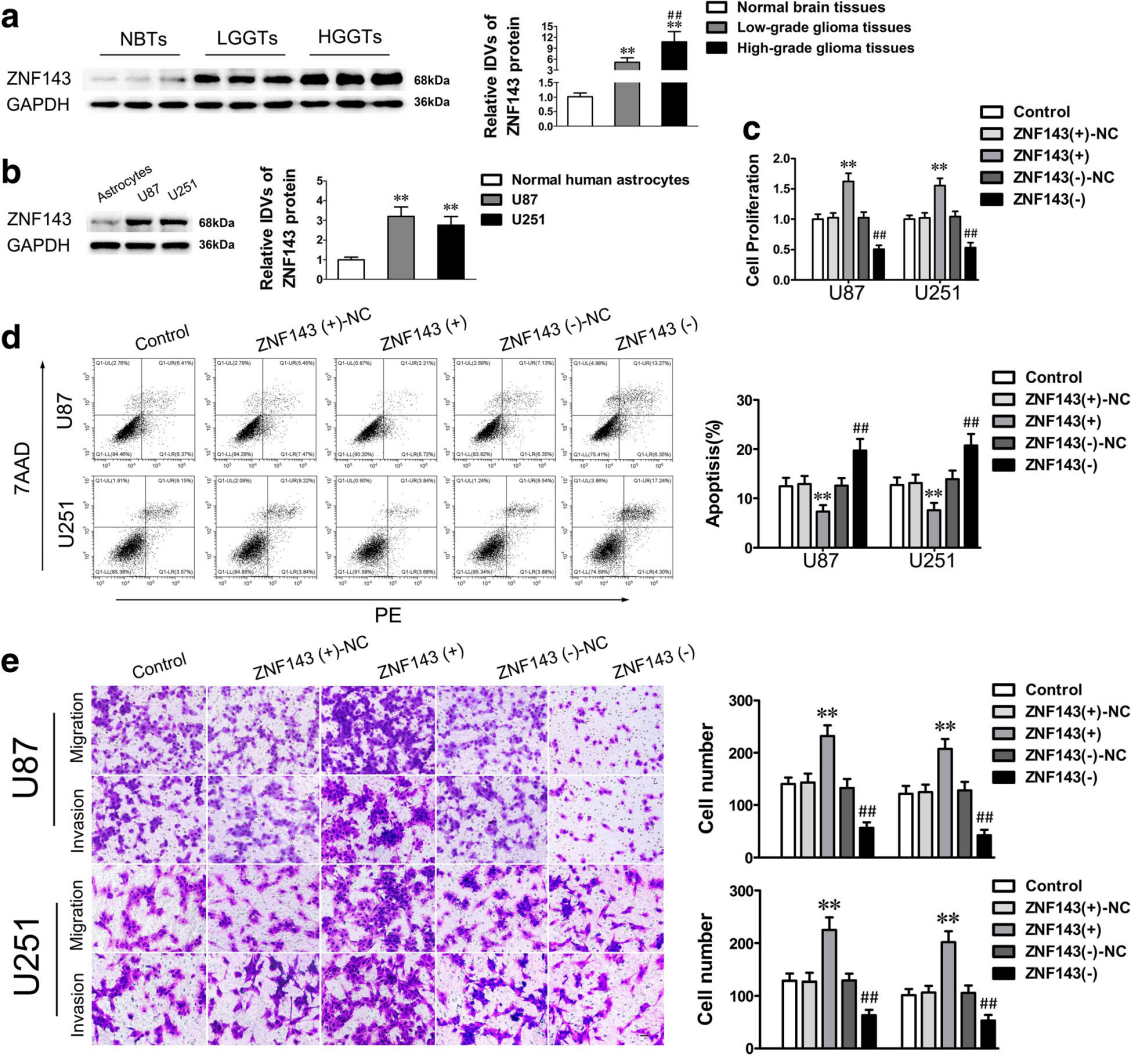
ZNF143 was upregulated and functioned as an oncogene in glioma cells. **a.** ZNF143 was overexpressed in glioma tissues. Data are presented as the mean ± SD (*n* = 6, NBTs; *n* = 18, Low-grade glioma tissues; *n* = 25, High-grade glioma tissues). ^**^P < 0.01 vs. Normal brain tissues. ^##^*P* < 0.01 vs. Low-grade glioma tissues. **b.** The ZNF143 expression was upregulated in glioma cells. Data are presented as the mean ± SD (*n* = 5, each group). ^**^P < 0.01 vs. NHA. **c-e.** CCK-8 assay, flow cytometry analysis and migration and invasion assays were applied to measure the biological behaviors of glioma cells treated with ZNF143 overexpression or knockdown. Data represented mean ± SD (*n* = 5, each group). ^**^P < 0.01 vs. ZNF143 (+)-NC group (negative control); ^##^*P* < 0.01 vs. ZNF143 (−)-NC group (negative control). Scale bar of migration and invasion assays represent 40 μm

A bioinformatics database (TargetScan) was used to identify ZNF143 as a putative downstream target of miR-590-3p. To affirm this hypothesis, the function of ZNF143 in glioma was firstly inspected. Western blot analysis showed that ZNF143 was overexpressed in glioma tissues and cells compared with NBTs and NHA (Fig. 4a and b). Moreover, the expression level of ZNF143 was positively correlated with the pathological grade of glioma (Fig. 4a). Importantly, knockdown of ZNF143 significantly impaired the malignant biological behavior of glioma cells (Fig. 4c–e). These results suggested that ZNF143 exerted oncogenic functions in glioma cells.

### MiR-590-3p negatively modulates ZNF143 expression by directly targeting its 3′ UTR

**Fig. 5:**
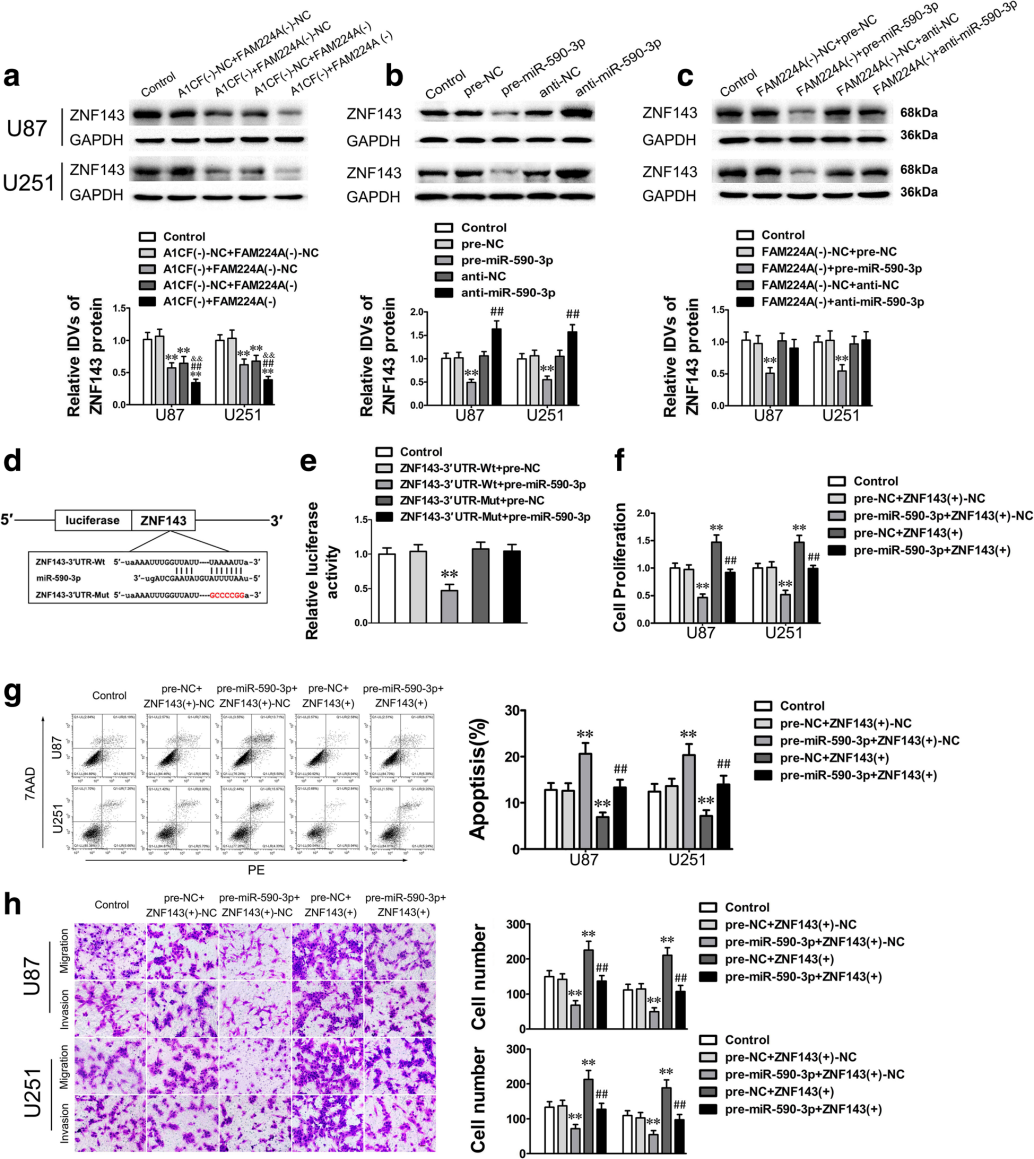
ZNF143 was a target of miR-590-3p and could rescue the tumor-suppressive impacts of miR-590-3p. **a**. The ZNF143 expression of glioma cells after A1CF and FAM224A knockdown was showed. **b.** The ZNF143 expression of glioma cells treated with altering miR-590-3p expression was illustrated. **c.** The ZNF143 expression of glioma cells co-transfected with sh-FAM224A and anti-miR-590-3p was exhibited. **d.** The potential miR-590-3p binding sites in ZNF143 3′ UTR and the designed mutant sequence were indicated. **e.** Relative luciferase activity was evaluated after cells were co-transfected with pre-miR-590-3p and ZNF143–3′ UTR-Wt or ZNF143–3′ UTR-Mut. Data were presented as the mean ± SD (*n* = 5, each group). ^**^P < 0.01 vs. ZNF143–3′ UTR Wt + pre-NC group. **f-h.** CCK-8 assay, flow cytometry analysis and migration and invasion assays were conducted to determine the biological behaviors of glioma cells co-transfected with miR-590-3p and ZNF143. Data represented mean ± SD (*n* = 5, each group). ^**^P < 0.01 vs. pre-NC + ZNF143 (+)-NC group, ^##^*P* < 0.01 vs. pre-miR-590-3p + ZNF143 (+)-NC group. Scale bar of migration and invasion assays assay represent 40 μm

The results above revealed that ZNF143 acted as an oncogene in glioma; however, the potential role of ZNF143 in A1CF and FAM224A induced malignant progression of glioma cells remained to be defined. Firstly, the ZNF143 expression of glioma cells treated with altering levels of A1CF, FAM224A or miR-590-3p expression was examined. As shown in Fig. 5a and b, ZNF143 expression was obviously inhibited by A1CF and FAM224A knockdown, or miR-590-3p overexpression. Moreover, the downregulation of ZNF143 expression in sh-FAM224A glioma cells was reversed following miR-590-3p knockdown (Fig. 5c). Subsequently, a dual-luciferase assay was performed to verify whether ZNF143 was a downstream target of miR-590-3p. The results showed that luciferase activity in the ZNF143-Wt + pre-miR-590-3p group declined considerably when compared with the ZNF143-Wt + pre-NC group, whereas no obvious alteration was found in the ZNF143-Mut groups (Fig. 5d and e). The evidence above demonstrated that miR-590-3p could directly bind to the specific sequence of the ZNF143 3′ UTR.

### ZNF143 reverses the miR-590-3p-induced inhibitory functions in glioma cells

To investigate whether ZNF143 mediates the inhibitory effects of miR-590-3p, the biological behaviors of U87 and U251 cells were assessed by CCK-8 assay, flow cytometry analysis, and migration and invasion assays after treatment with miR-590-3p and ZNF143 upregulation. As shown in Fig. 5f–h, the proliferation, migration and invasion abilities of glioma cells were elevated, while the percentages of apoptotic cells were diminished in the pre-miR-590-3p + ZNF143 (+) group versus the pre-miR-590-5p + ZNF143 (+)-NC group. These results indicated that miR-590-3p suppressed the malignant progression of glioma cells via downregulating ZNF143 expression.

### Oncogenes ASAP3 and MYB are involved in ZNF143-mediated modulation of malignant progression of glioma cells

**Fig. 6:**
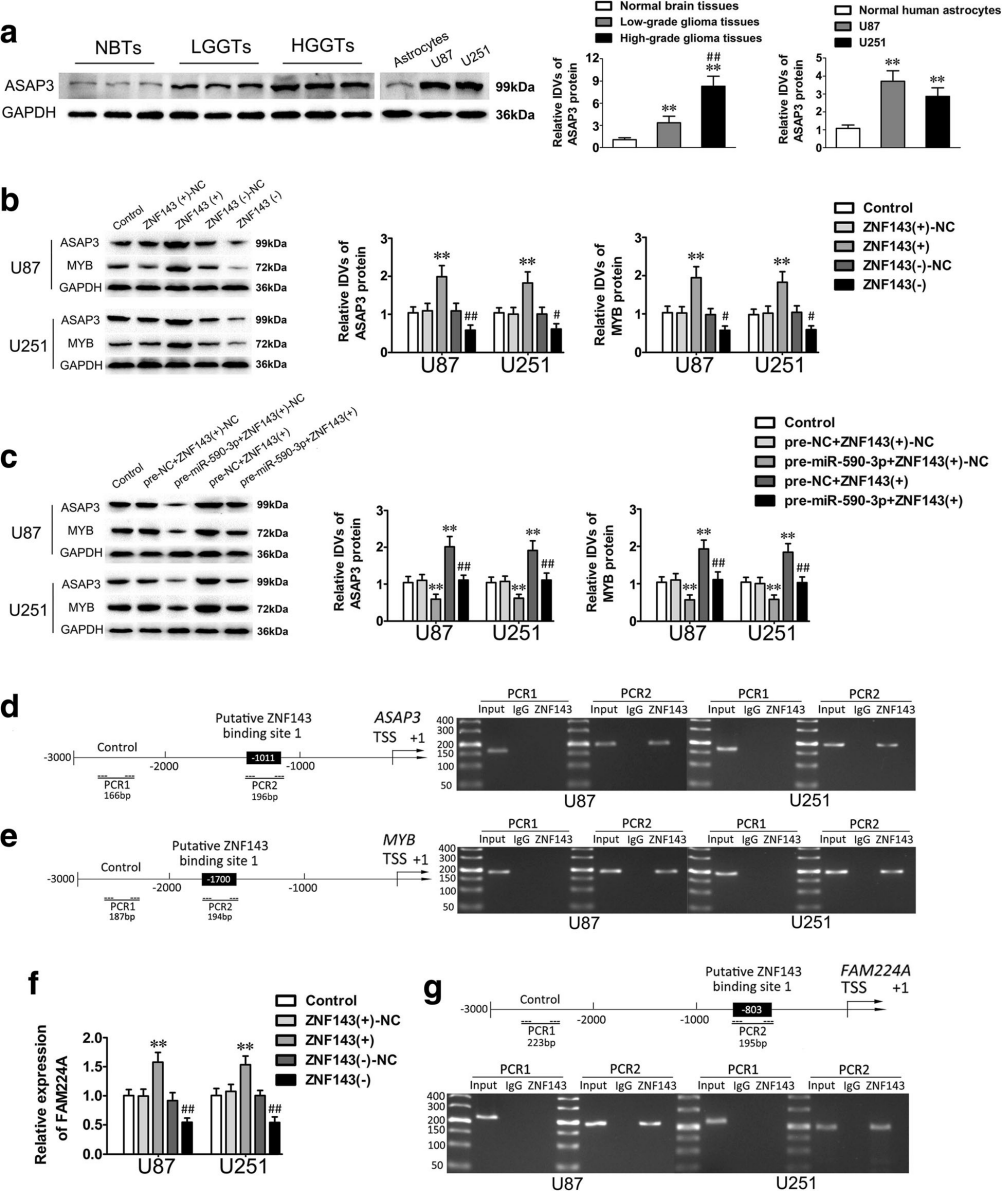
ASAP3 and MYB were downstream targets of ZNF143, moreover, FAM224A expression was activated by ZNF143. **a**. The expression levels of ASAP3 were upregulated in glioma tissues (left) and cells (right). Left: Data are presented as the mean ± SD (*n* = 6, NBTs; *n* = 18, Low-grade glioma tissues; *n* = 25, High-grade glioma tissues). ^**^P < 0.01 vs. Normal brain tissues. ^##^*P* < 0.01 vs. Low-grade glioma tissues. Right: Data are presented as the mean ± SD (*n* = 5, each group). ^**^P < 0.01 vs. NHA. **b-c.** Weastern blot assay showed that the expression of ASAP3 and MYB were regulated by miR-590-3p and ZNF143. **d-e.** ChIP assay demonstrated that ZNF143 could promote ASAP3 and MYB expression by binding to their promoters in glioma cells. **f-g.** qRT-PCR and ChIP assays confirmed that ZNF143 could directly activate the promoter of FAM224A and upregulated its expression in glioma cells

Based on data derived from bioinformatics databases (DBTSS HOME and JASPAR), we speculated that ASAP3 and MYB proto-oncogene (MYB) were putative downstream targets of ZNF143. To test our hypothesis, ASAP3 expression was firstly detected. As shown in Fig. 6a, ASAP3 was dramatically upregulated in glioma tissues and cells compared with NBTs and NHA. In addition, the expression level was positively correlated with the pathological grade of glioma (Fig. 6a). Remarkably, ASAP3 knockdown significantly inhibited the malignant biological behaviors of glioma cells such as cell proliferation, migration and invasion, while facilitating cell apoptosis (Additional file 4: Figure S4a–c). This evidence inferred that ASAP3 served as an oncogene in glioma cells.

In our further studies, the ASAP3 and MYB expression of glioma cells treated with altering miR-590-3p and ZNF143 expression levels was detected. As shown in Fig. 6b, ZNF143 knockdown obviously reduced ASAP3 and MYB expression. Furthermore, overexpression of ZNF143 reversed the inhibitory effects of overexpressed miR-590-3p on glioma cell progression via upregulating ASAP3 and MYB expression (Fig. 6c).

The results of a ChIP assay provided insight into the interaction and putative binding site of ASAP3 and MYB. According to the database DBTSS HOME, the promoter sequences of ASAP3 and MYB were established. The predicted binding sites of the ASAP3 and the MYB promoter were observed at − 1011 in the ASAP3 transcription start site (TSS) and at − 1700 in the MYB TSS. As the ChIP results corroborated, there was a direct interaction between ZNF143 and ASAP3 or MYB, while no association was found between ZNF143 and the control regions (Fig. 6d and e). Overall, these findings indicated that miR-590-3p could impede ASAP3 and MYB expression by downregulating ZNF143 in glioma cells.

### ZNF143 feedback promotes FAM224A expression through binding to its promoter

Using bioinformatic databases (DBTSS HOME and JASPAR), the potential ZNF143 binding region of the FAM224A promoter was identified at − 803 in the FAM224A TSS. To confirm our speculation, the expression of FAM224A was firstly detected in glioma cells treated with altered expression levels of ZNF143. The qRT-PCR results revealed an obvious increase in FAM224A expression when ZNF143 was upregulated (Fig. 6f). Furthermore, a ChIP assay exhibited that ZNF143 could directly bind to the FAM224A promoter (Fig. 6g).

### Knockdown of A1CF and FAM224A combined with overexpression of miR-590-3p inhibited tumor xenograft growth and exhibited the highest survival rates

**Fig. 7:**
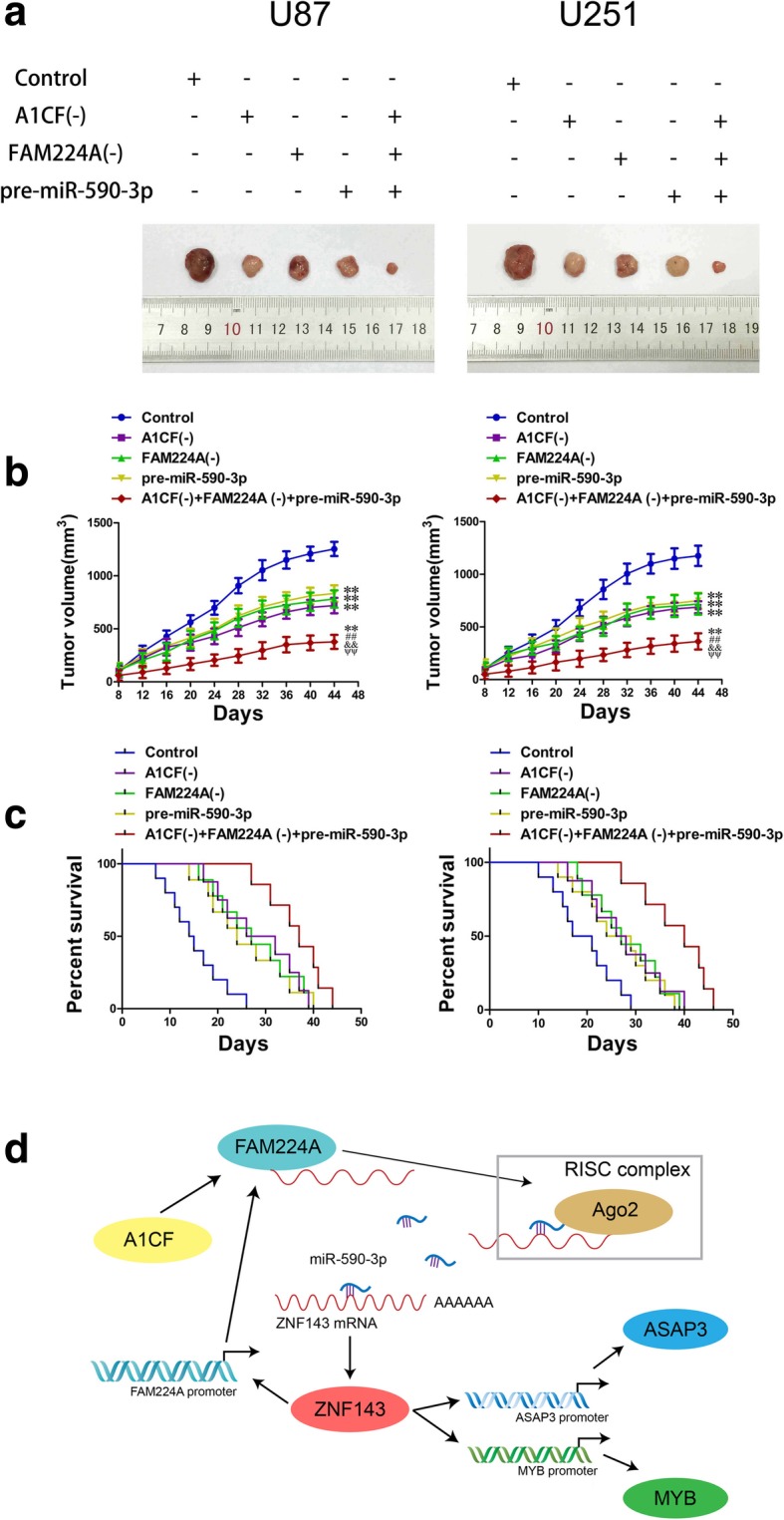
In vivo study. **a**. The sample tumors from respective groups were presented. **b**. Tumor xenograft growth curves in subcutaneous implantation assay were displayed. Data represent mean ± SD (n = 10, each group). ^**^P < 0.01 vs. Control group, ^##^*P* < 0.01 vs. A1CF (−) group, ^&&^*P* < 0.01 vs. FAM224A (−) group, ^**ΨΨ**^*P* < 0.01 vs. pre-miR-590-3p group. **c.** Survival curves from representative nude mice injected into the right striatum in orthotopic inoculations assay were exhibited. (log-rank test, *P* < 0.01, A1CF (−), FAM224A (−), pre-miR-590-3p and A1CF (−) + FAM224A (−) + pre-miR-590-3p groups versus Control group). **d**. The schematic diagram underlying the mechanism of A1CF/FAM224A/miR-590-3p/ZNF143 positive feedback loop in glioma cells

In vivo experiments were conducted to further confirm the above findings. The results showed that the tumor volumes in the A1CF (−), FAM224A (−), pre-miR-590-3p and A1CF (−) + FAM224A (−) + pre-miR-590-3p groups were smaller than those in the control group. Additionally, knockdown of A1CF and FAM224A combined with miR-590-3p overexpression resulted in the smallest tumor sizes among all of the groups (Fig. 7a and b). As shown in Fig. 7c, survival analysis in orthotopic inoculations assay revealed that mice in the A1CF (−), FAM224A (−), pre-miR-590-3p and A1CF (−) + FAM224A (−) + pre-miR-590-3p groups had longer survival times than the mice in the control group, with mice treated with A1CF (−) + FAM224A (−) + pre-miR-590-3p having the longest survival times.

## Discussion

In this study, we confirmed that A1CF and FAM224A were overexpressed in glioma tissues and cell lines. Knockdown of A1CF significantly suppressed the biological behaviors of glioma cells via destabilizing FAM224A. Conversely, miR-590-3p functioned as a tumor-suppressor in glioma. MiR-590-3p was negatively modulated by the miRNA sponge-like role of FAM224A and they were both involved in an RNA-induced silencing complex. In addition, ZNF143 exerted an oncogenic role through activating ASAP3 and MYB expression. MiR-590-3p could bind to the ZNF143 3′ UTR and repress the expression of ASAP3 and MYB by negative modulation of ZNF143, thereby hindering the malignant progression of glioma cells. Strikingly, FAM224A expression could be promoted via feedback by ZNF143 through activating the FAM224A promoter. Finally, A1CF and FAM224A inhibition combined with overexpression of miR-590-3p predominantly suppressed tumor growth, as well as elongating the survival time of nude mice.

Accumulating evidence indicates that dysregulation of RBPs is intimately related to the malignant progression of various tumors. For example, overexpression of HuR significantly suppresses cell apoptosis in colorectal cancer [26]. RBP A1CF belongs to the heterogeneous nuclear ribonucleoprotein family and contributes key functions to multiple essential cellular processes [27]. Recent research showed that A1CF acts as an oncogene in breast cancer by enhancing the stability of Dickkopf1 [28]. LncRNAs are identified as an indispensable link in well-known carcinogenic and tumor-suppressive networks. They exert critical modulatory functions in the malignant progression of glioma. CRNDE and SBF2-AS1 are overexpressed and promote the malignant biological behaviors of glioma cells, while Gas5 plays a tumor-suppressive role in glioma cells [29–31]. Our present data revealed that A1CF and FAM224A were obviously upregulated in glioma tissues and cells. High expression levels of A1CF and FAM224A were intimately associated with the poor prognosis of glioma patients. In addition, depletion of A1CF or FAM224A markedly attenuated the proliferation, migration and invasion of glioma cells, whereas promoted cellular apoptosis. These results revealed, for the first time, that A1CF and FAM224A play an oncogenic role in glioma cells; however, the underlying mechanisms need to be investigated.

A growing number of studies have uncovered the interaction of RBPs and lncRNAs in a range of tumors. RBPs have been shown to upregulate lncRNAs expression by strengthening the stability of these RNAs [32–34]. For example, PABPC1 promotes the proliferation and metastasis of gallbladder cancer cells by enhancing PAGBC stability [34]. In our study, we discovered that overexpression of A1CF remarkably upregulated FAM224A expression in glioma cells. Moreover, knockdown of A1CF significantly decreased FAM224A expression by reducing the half-life of FAM224A, whereas nascent FAM224A was not impacted. These results illustrated that A1CF could upregulate FAM224A expression by strengthening its stability. Liu et al. [35] reported that TDP43 promoted the malignant progression of glioma cells by stabilizing SNHG12, which further supports our findings.

Numerous lines of evidence have confirmed the cross-regulation between lncRNAs and miRNAs. LncRNAs may serve as competing endogenous RNAs to modulate the expression and functions of miRNAs, and are therefore known as “miRNA sponges” [36, 37]. To understand the potential oncogenic mechanisms of FAM224A in glioma cells, we used a bioinformatics database (Starbase) to identify miR-590-3p as an target of FAM224A. MiR-590-3p exerts crucial biological functions in multifarious human tumors [38–40]. Our results exhibited that miR-590-3p expression was significantly downregulated in glioma and restoration of miR-590-3p dramatically restrained the malignant evolution of glioma cells, indicating that miR-590-3p was a tumor-suppressor in glioma cells. Consistent with our findings, miR-590-3p was reported to be expressed at low levels and participate in the tumor-suppressive processes induced by EMAP-II and temozolomide in glioma cells [41]. By dual-luciferase and RIP assays, we verified that FAM224A could bind to miR-590-3p and that they were both involved in an RNA-induced silencing complex. In addition, FAM224A knockdown upregulated miR-590-3p expression, whereas miR-590-3p overexpression decreased FAM224A expression. Furthermore, miR-590-3p knockdown rescued the tumor-inhibitory effects of anti-FAM224A on glioma cells. These results suggested that there exists a reciprocal repression feedback loop between FAM224A and miR-590-3p. Similarly, the “miRNA sponge” effect of lncRNAs has also been affirmed by other research into glioma. SOX2OT impairs the malignant progression of glioma stem cells by upregulating the expression of miR-194-5p and miR-122 [42]. TUG1 maintains the stemness of glioma stem cells by acting as a molecular sponge of miR-145 [43]. OIP5-AS1 and miR-367-3p reciprocally regulate their expression, influencing the biological behaviors of glioma cells [1]. Remarkably, in vivo experiments elucidated that the nude mice in the A1CF (−) + FAM224A (−) + pre-miR-590-3p group exhibited the minimum tumor sizes and longest survival times. Collectively, knockdown of A1CF could increase miR-590-3p expression via downregulating FAM224A; however, the molecular mechanisms of miR-590-3p-induced tumor-suppressive functions still remain unknown.

MiRNAs can post-transcriptionally regulate the expression of downstream genes via binding to their 3′ UTRs in tumors. Based on data in a bioinformatics database (TargetScan), we speculated that ZNF143 might be a downstream modulatory target of miR-590-3p. Dual-luciferase and western blot assays certified that miR-590-3p could directly target the ZNF143 3′ UTR and negatively modulate its expression. An earlier study also reported that ZNF143 was negatively regulated by miR-590-3p at the post-transcriptional level in teratocarcinoma cells, which upheld our findings [44]. Previous studies have demonstrated that ZNF143 plays carcinogenic roles in diverse tumors, such as lung adenocarcinoma, gastric cancer and colon cancer [22, 45, 46]. Our data showed that ZNF143 was significantly upregulated in glioma tissues and cell lines. Downregulation of ZNF143 expression markedly impeded the malignant biological behaviors of glioma cells, affirming the oncogenic role of ZNF143 in glioma cells. Additionally, inhibition of A1CF and FAM224A obviously reduced ZNF143 expression in glioma cells, and upregulation of ZNF143 reversed the prohibitive effects on glioma cells induced by miR-590-3p overexpression. These findings provided evidence that ZNF143 engages in the A1CF-mediated modulation of the malignant progression of glioma cells.

Under most circumstances, transcription factors can activate target genes through binding to their promoter regions. Using bioinformatics databases (DBTSS HOME and JASPAR), we found that the promoter regions of ASAP3 and MYB contained potential binding sequences of ZNF143, inferring that ASAP3 and MYB might be downstream targets of ZNF143. ASAP3 plays a key regulatory role in cell migration, invasion and metastasis of various tumors [47–49]. In our study, the expression levels of ASAP3 were remarkably elevated in glioma tissues and cells. In addition, knockdown of ASAP3 dramatically hindered the biological behaviors of glioma cells. Consistent with our results, the oncogenic functions of ASAP3 have also been verified in lung cancer as well as breast cancer [49, 50]. MYB has been proven to be highly expressed in glioma cells and promotes cell proliferation, migration and invasion [51, 52]. To validate our assumption, we firstly detected the expression of ASAP3 and MYB in glioma cells treated with altered expression levels of miR-590-3p and ZNF143 expression. Our results suggested that overexpression of miR-590-3p obviously reduced the expression of ASAP3 and MYB, thereby restraining the biological behaviors of glioma cells. Whereas, overexpression of ZNF143 reversed the miR-590-3p-mediated tumor-suppressive effects through elevating ASAP3 and MYB expression. Then, ChIP experiments corroborated that ZNF143 directly targets the specific binding sites in the ASAP3 and MYB promoter regions. Therefore, ASAP3 and MYB are involved in mediating the regulatory effects of miR-590-3p on glioma cell progression, after being activated by ZNF143.

Emerging evidences suggests that transcription factors upregulate lncRNAs expression by activating their promoter regions and forming a positive feedback loop. For example, HCP5 enhances the biological behaviors of glioma cells by upregulating RUNX1, while RUNX1 also activates HCP5 expression through binding to its promoter region, forming a positive feedback loop [12]. Similarly, SNHG12 could upregulate SOX5 expression and exert oncogenic functions in glioma cells, while SOX5 increases SNHG12 expression by activating its promoter [35]. In the present study, we discovered the putative binding sequence of ZNF143 in the promoter region of FAM224A based on bioinformatics databases (DBTSS HOME and JASPAR). Furthermore, a ChIP assay corroborated that ZNF143 directly targets the specific binding site in the FAM224A promoter. What’s more, overexpression of ZNF143 remarkably increased FAM224A expression. These results explicated the existence of a positive feedback loop between FAM224A and ZNF143.

## Conclusions

In summary, this study revealed that A1CF can upregulate the expression of FAM224A via strengthening its stability, which in turn, influenced the negative modulation of miR-590-3p on transcription factor ZNF143. Furthermore, ZNF143 promoted the malignant biological behaviors of glioma cells by stimulating ASAP3 and MYB expression. ZNF143 can activate FAM224A expression through binding to its promoter region, forming a positive feedback loop. In conclusion, the A1CF/FAM224A/miR-590-3p/ZNF143 positive feedback loop acts as a critical regulator in the malignant progression of glioma cells, providing a novel molecular target for glioma therapy.

## Additional files

Additional file 1: **Figure S1.** LncRNAs microarrays data in glioma U87 and U251 cells. LncRNAs gene expression profiles were obtained from glioma U87 and U251 cell samples as indicated. (JPG 546 kb)

Additional file 2: **Figure S2.** A1CF and FAM224A played an oncogenic role in glioma cells. **a**-**c**. CCK-8 assay, flow cytometry analysis, migration and invasion assays were appled to investigate the effects of A1CF on biological behaviors of glioma cells. **P < 0.01 vs. A1CF(+)-NC group (negative control); ##*P* < 0.01 vs. A1CF(-)-NC group (negative control). **d**-**f**. CCK-8 assay, flow cytometry analysis, migration and invasion assays were conducted to determine the functions of FAM224A in glioma cells. **P < 0.01 vs. FAM224A (+)-NC group (negative control); ##*P* < 0.01 vs. FAM224A (-)-NC group (negative control). Scale bar of migration and invasion assays represent 40 μm. (JPG 13147 kb)

Additional file 3: **Figure S3.** MiR-590-3p exerted tumor-suppressive function in glioma cells. **a**-**c**. CCK-8 assay, flow cytometry analysis, migration and invasion assays were utilized to determine the influences of miR-590-3p expression alteration on biological behaviors of glioma cells. Data are presented as the mean ± SD (n = 5, each group). **P < 0.01 vs. pre-NC group (negative control); ##*P* < 0.01 vs. anti-NC group (negative control). Scale bar of migration and invasion assays represent 40 μm. (JPG 6180 kb)

Additional file 4: **Figure S4.** ASAP3 played an oncogenic role in glioma cells. **a**-**c**. CCK-8 assay, flow cytometry analysis and migration and invasion assays were used to measure the biological behaviors of glioma cells treated with ASAP3 overexpression or knockdown. Data are presented as the mean ± SD (n = 5, each group). **P < 0.01 vs. ASAP3(+)-NC group (negative control); ##*P* < 0.01 vs. ASAP3(−)-NC group (negative control). Scale bar of migration and invasion assays represent 40 μm. (JPG 4026 kb)

Additional file 5: **Figure S5.** The transfection efficacy was detected by qRT-PCR or western blot **a**. Western blot was used to examine the expression of A1CF in glioma cells treated with altering A1CF expression. Data represented mean ± SD (n=5, each group). **P < 0.01 vs. A1CF(+)-NC group; ##*P* < 0.01 vs. A1CF(-)-NC group. **b**. qRT-PCR was used to detect the expression of FAM224A in glioma cells treated with altering FAM224A expression. Data represented mean ± SD (n=5, each group). **P < 0.01 vs. FAM224A(+)-NC group; ##*P* < 0.01 vs. FAM224A(-)-NC group. **c**. The ZNF143 expression of glioma cells after ZNF143 overexpression or knockdown was showed. Data represented mean ± SD (n = 5, each group). **P < 0.01 vs. ZNF143(+)-NC group; ##*P* < 0.01 vs. ZNF143(−)-NC group. **d**. The miR-590-3p expression of glioma cells transfected with miR-590-3p agomir or antagomir was displayed. Data are presented as the mean ± SD (n = 5, each group). **P < 0.01 vs. pre-NC group; ##*P* < 0.01 vs. anti-NC group. **e**. The ASAP3 expression of glioma cells after ASAP3 overexpression or knockdown was examined. Data are presented as the mean ± SD (n = 5, each group). **P < 0.01 vs. ASAP3(+)-NC group; ##*P* < 0.01 vs. ASAP3(−)-NC group; #*P* < 0.05 vs. ASAP3(−)-NC group. (JPG 1335 kb)

Additional file 6: Supplementary Tables. (DOC 56 kb)

## Abbreviations

3′ UTR: 3′ untranslated region
A1CF: APOBEC1 complementation factor
APOB: Apolipoprotein B
ASAP3: ArfGAP with SH3 domain, ankyrin repeat and PH domain 3
CCK-8: Cell counting kit-8
ChIP: Chromatin immunoprecipitation assay
FAM224A: Family with sequence similarity 224 member A
lncRNAs: Long non-coding RNAs
MYB: MYB proto-oncogene
NBTs: Normal brain tissues
NHA: Normal human astrocyte
qRT-PCR: Quantitative RT-PCR
RBPs: RNA binding proteins
RIP: RNA Immunoprecipitation
SD: Standard deviation
SDS-PAGE: Sodium dodecyl sulfate polyacrylamide gel electropheresis
ZNF143: Zinc finger protein 143
