# Supplementary figures and images for: Inhibition of the aberrant A1CF-FAM224A-miR-590-3p-ZNF143 positive feedback loop attenuated malignant biological behaviors of glioma cells
Source: J Exp Clin Cancer Res. 2019 Jun 11;38:248. doi: 10.1186/s13046-019-1200-5 (PMC6558706; doi:10.1186/s13046-019-1200-5)

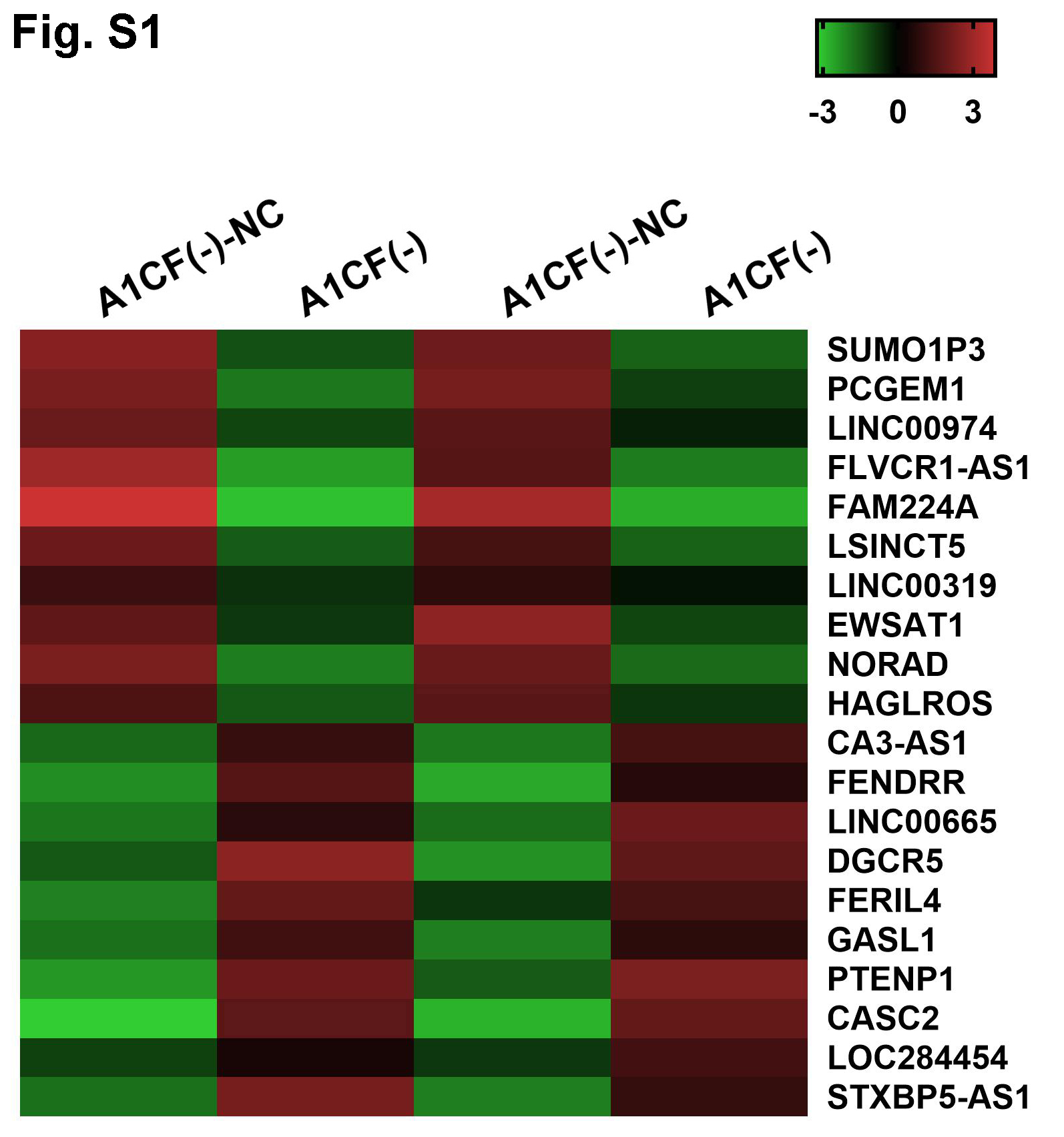

Supplement: Supplementary file 1 — Figure S1. LncRNAs microarrays data in glioma U87 and U251 cells. LncRNAs gene expression profiles were obtained from glioma U87 and U251 cell samples as indicated. (JPG 546 kb) [file 13046_2019_1200_MOESM1_ESM.jpg]

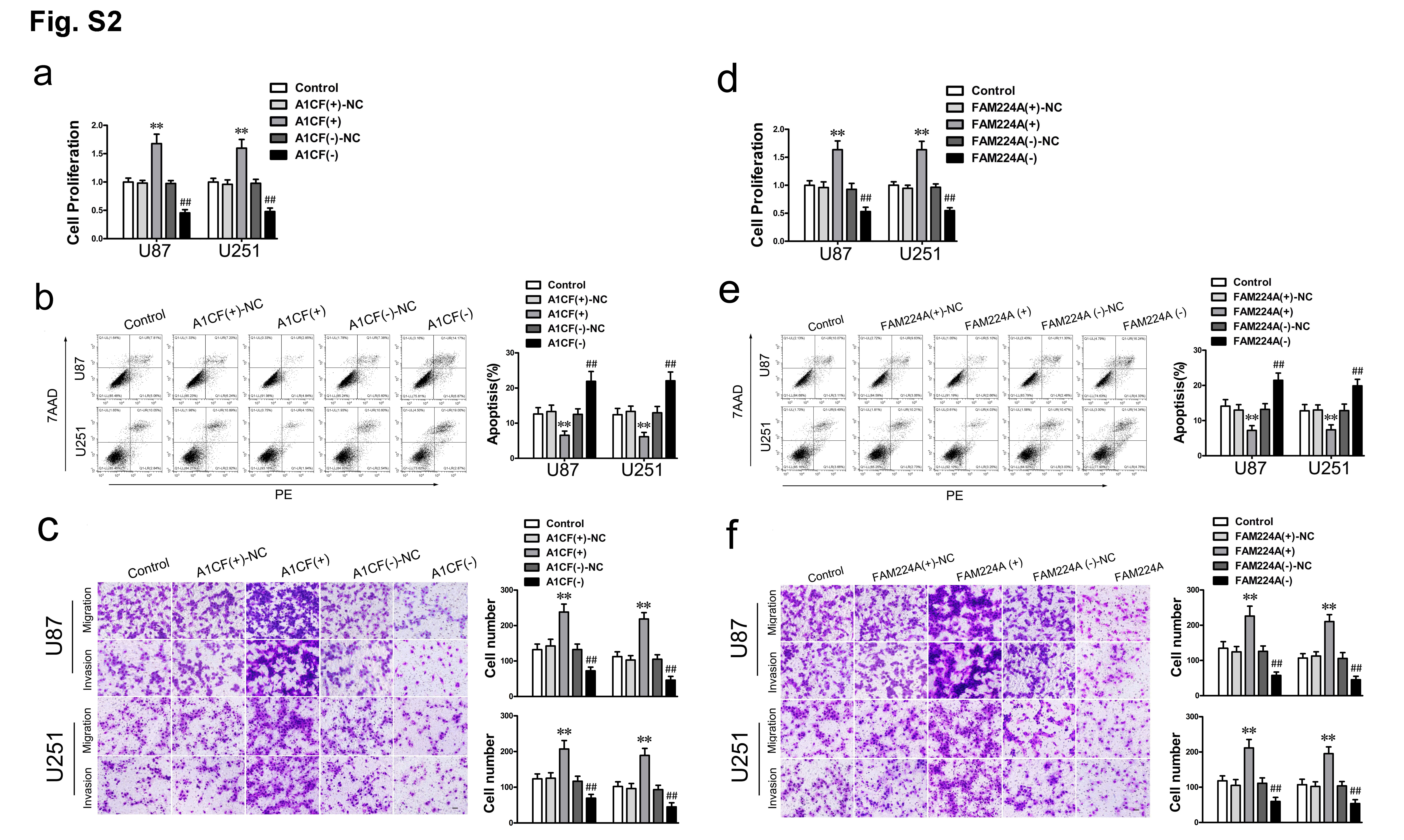

Supplement: Supplementary file 2 — Figure S2. A1CF and FAM224A played an oncogenic role in glioma cells. a-c. CCK-8 assay, flow cytometry analysis, migration and invasion assays were appled to investigate the effects of A1CF on biological behaviors of glioma cells. **P < 0.01 vs. A1CF(+)-NC group (negative control); ##P < 0.01 vs. A1CF(-)-NC group (negative control). d-f. CCK-8 assay, flow cytometry analysis, migration and invasion assays were conducted to determine the functions of FAM224A in glioma cells. **P < 0.01 vs. FAM224A (+)-NC group (negative control); ##P < 0.01 vs. FAM224A (-)-NC group (negative control). Scale bar of migration and invasion assays represent 40 μm. (JPG 13147 kb) [file 13046_2019_1200_MOESM2_ESM.jpg]

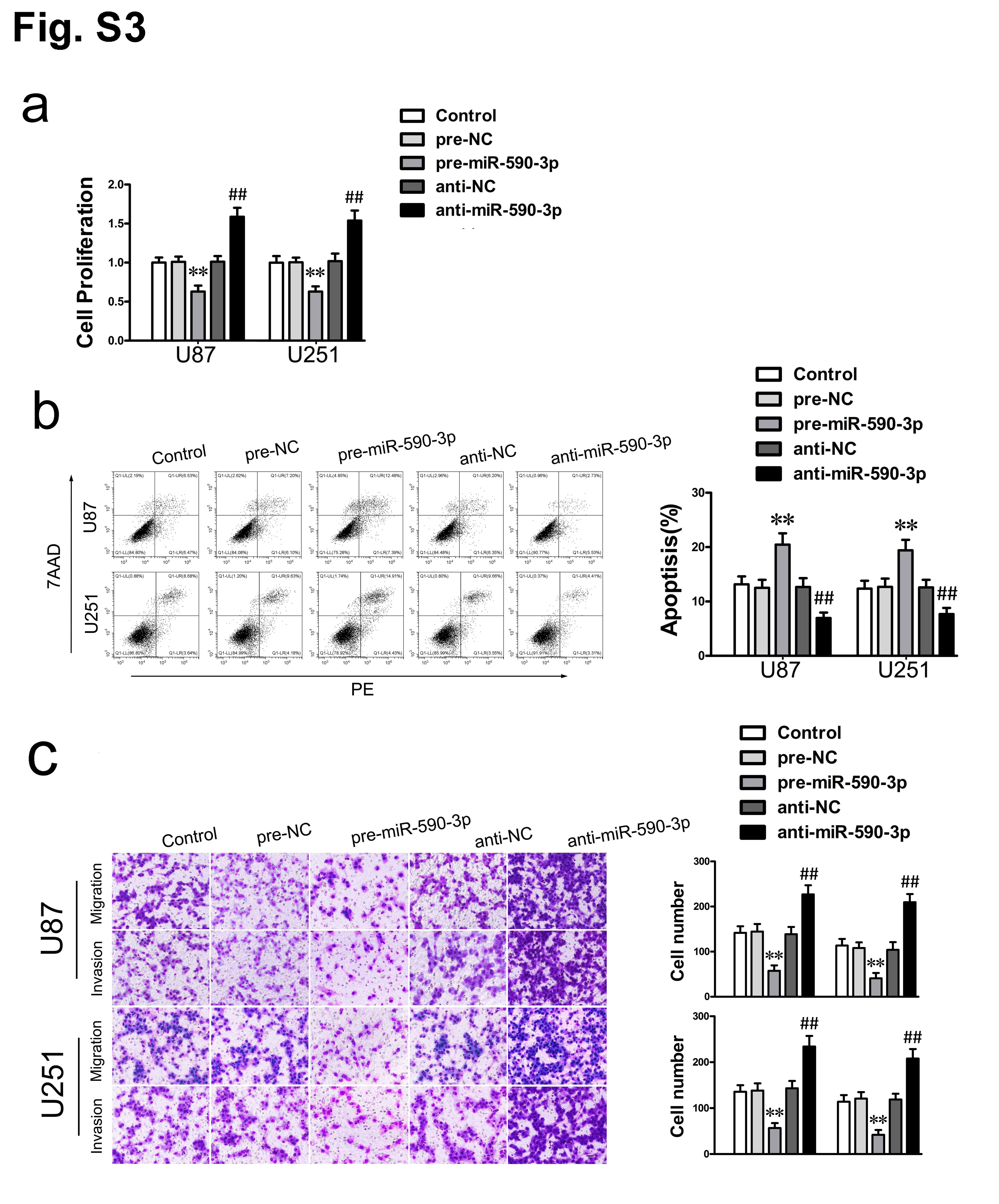

Supplement: Supplementary file 3 — Figure S3. MiR-590-3p exerted tumor-suppressive function in glioma cells. a-c. CCK-8 assay, flow cytometry analysis, migration and invasion assays were utilized to determine the influences of miR-590-3p expression alteration on biological behaviors of glioma cells. Data are presented as the mean ± SD (n = 5, each group). **P < 0.01 vs. pre-NC group (negative control); ##P < 0.01 vs. anti-NC group (negative control). Scale bar of migration and invasion assays represent 40 μm. (JPG 6180 kb) [file 13046_2019_1200_MOESM3_ESM.jpg]

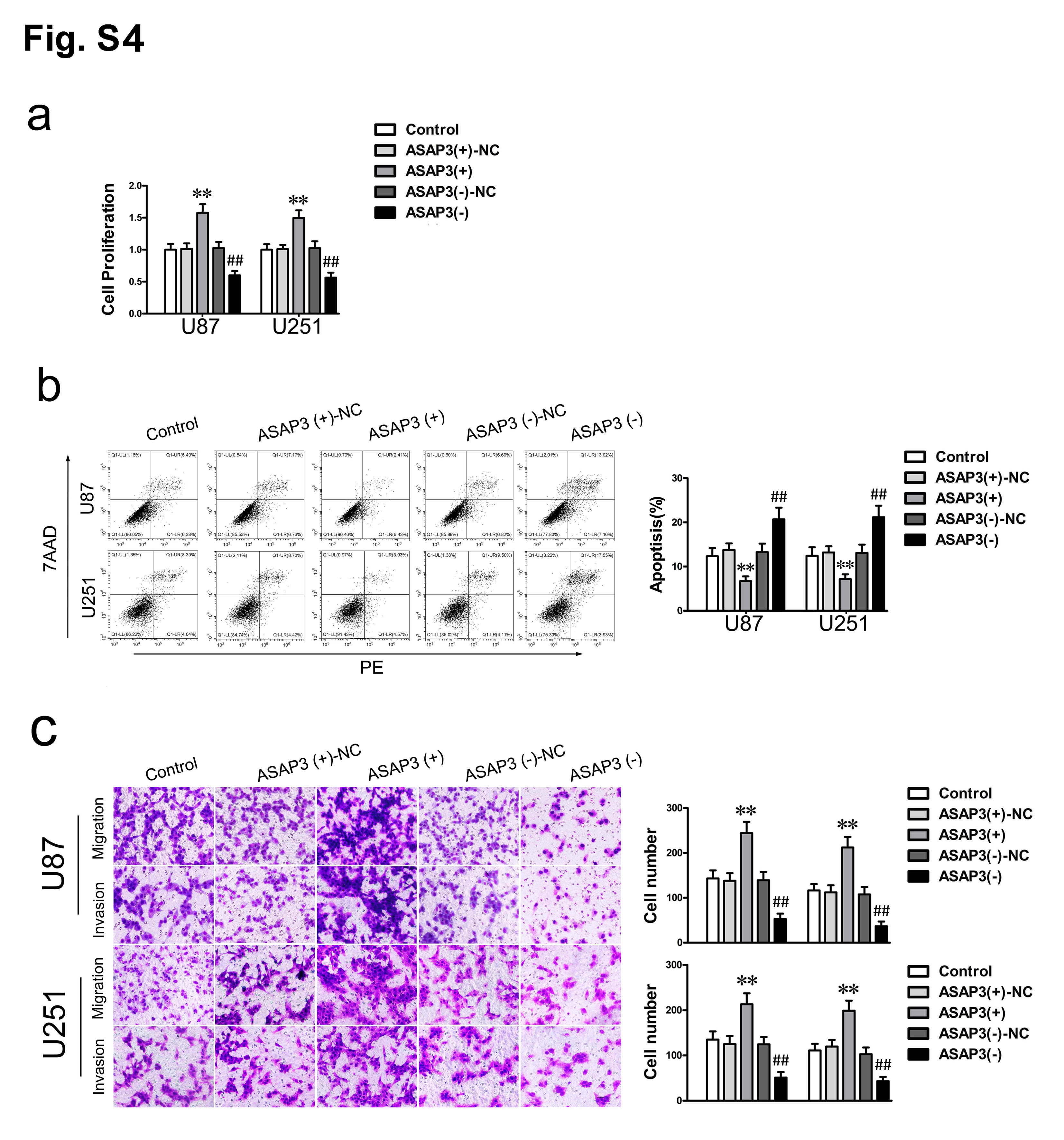

Supplement: Supplementary file 4 — Figure S4. ASAP3 played an oncogenic role in glioma cells. a-c. CCK-8 assay, flow cytometry analysis and migration and invasion assays were used to measure the biological behaviors of glioma cells treated with ASAP3 overexpression or knockdown. Data are presented as the mean ± SD (n = 5, each group). **P < 0.01 vs. ASAP3(+)-NC group (negative control); ##P < 0.01 vs. ASAP3(−)-NC group (negative control). Scale bar of migration and invasion assays represent 40 μm. (JPG 4026 kb) [file 13046_2019_1200_MOESM4_ESM.jpg]

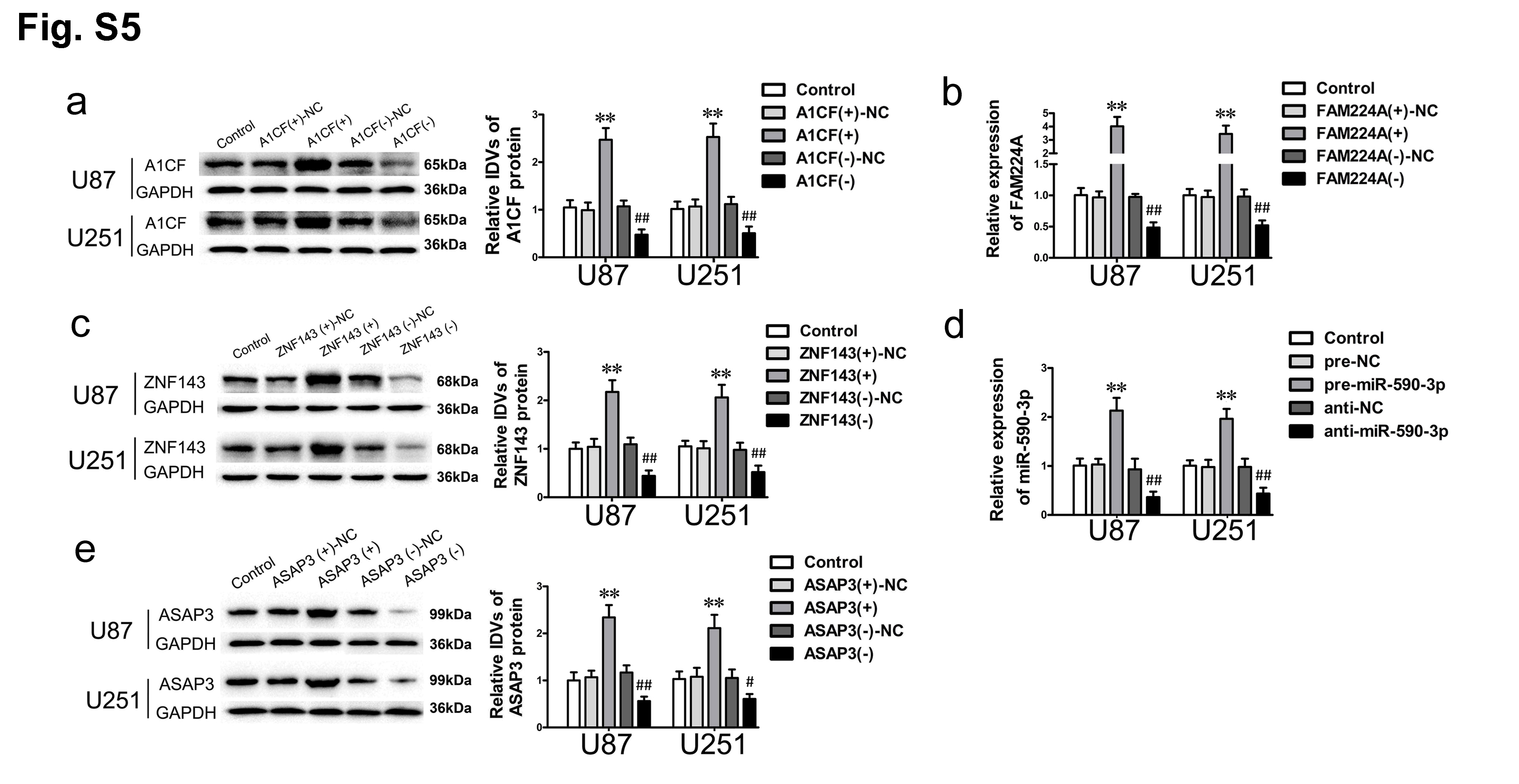

Supplement: Supplementary file 5 — Figure S5. The transfection efficacy was detected by qRT-PCR or western blot a. Western blot was used to examine the expression of A1CF in glioma cells treated with altering A1CF expression. Data represented mean ± SD (n=5, each group). **P < 0.01 vs. A1CF(+)-NC group; ##P < 0.01 vs. A1CF(-)-NC group. b. qRT-PCR was used to detect the expression of FAM224A in glioma cells treated with altering FAM224A expression. Data represented mean ± SD (n=5, each group). **P < 0.01 vs. FAM224A(+)-NC group; ##P < 0.01 vs. FAM224A(-)-NC group. c. The ZNF143 expression of glioma cells after ZNF143 overexpression or knockdown was showed. Data represented mean ± SD (n = 5, each group). **P < 0.01 vs. ZNF143(+)-NC group; ##P < 0.01 vs. ZNF143(−)-NC group. d. The miR-590-3p expression of glioma cells transfected with miR-590-3p agomir or antagomir was displayed. Data are presented as the mean ± SD (n = 5, each group). **P < 0.01 vs. pre-NC group; ##P < 0.01 vs. anti-NC group. e. The ASAP3 expression of glioma cells after ASAP3 overexpression or knockdown was examined. Data are presented as the mean ± SD (n = 5, each group). **P < 0.01 vs. ASAP3(+)-NC group; ##P < 0.01 vs. ASAP3(−)-NC group; #P < 0.05 vs. ASAP3(−)-NC group. (JPG 1335 kb) [file 13046_2019_1200_MOESM5_ESM.jpg]
