# Supplementary material for: Inhibition of the aberrant A1CF-FAM224A-miR-590-3p-ZNF143 positive feedback loop attenuated malignant biological behaviors of glioma cells
Source: J Exp Clin Cancer Res. 2019 Jun 11;38:248. doi: 10.1186/s13046-019-1200-5 (PMC6558706; doi:10.1186/s13046-019-1200-5)
Supplement: Supplementary file 6 — Supplementary Tables. (DOC 56 kb) [file 13046_2019_1200_MOESM6_ESM.doc]

| Characteristics | A1CF  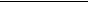 Low High | χ^2^ *P* value |
| --- | --- | --- |
| Age (y) |  | 1.685 0.1943 |
| ≤45 | 9 11 |  |
| ＞45 | 6 17 |  |
| Gender |  | 0.431 0.5116 |
| Male | 8 12 |  |
| Female | 7 16 |  |
| WHO grade  I grade  II grade | 6 2  7 3 | 19.061 0.0003^**^ |
| III grade  IV grade | 1 10  1 13 |  |

**Table S1. Correlation between clinicopathological characteristics and A1CF expression in 43 glioma patients.**

A1CF median expression level was used as cut-off.

*P* value was obtained by Pearson chi-square test.

^**^*P* < 0.01.

| Characteristics | FAM224A  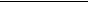Low High | χ^2^ *P* value |
| --- | --- | --- |
| Age (y) |  | 2.652 0.1034 |
| ≤45 | 11 9 |  |
| ＞45 | 7 16 |  |
| Gender |  | 1.018 0.3130 |
| Male | 10 10 |  |
| Female | 8 15 |  |
| WHO grade  I grade  II grade | 8 0  7 3 | 23.832 0.0001^**^ |
| III grade  IV grade | 2 9  1 13 |  |

**Table S2. Correlation between clinicopathological characteristics and FAM224A expression in 43 glioma patients.**

FAM224A median expression level was used as cut-off.

*P* value was obtained by Pearson chi-square test.

^**^*P* < 0.01.

**Table S3**. Sequences of shRNA template

| Gene |  | Sequence(5'->3') |
| --- | --- | --- |
| A1CF  FAM224A | Sence  Antisence  Sence  Antisence | CACCGGTCCAGCGCACAGGATATAGTTCAAGAGACTATATCCTGTGCGCTGGACCTTTTTTG  GATCCAAAAAAGGTCCAGCGCACAGGATATAGTCTCTTGAACTATATCCTGTGCGCTGGACC  CCGGCTCATGGACTCTGGACTGAACTCGAGTTCAGTCCAGAGTCCATGAGGTTTTTG  GATCCAAAAACCTCATGGACTCTGGACTGAACTCGAGTTCAGTCCAGAGTCCATGAGG |
| ZNF143 | Sence | CACCGCTACAAGAGTAACTGCTAAATTCAAGAGATTTAGCAGTTACTCTTGTAGCTTTTTTG |
|  | Antisence | GATCCAAAAAAGCTACAAGAGTAACTGCTAAATCTCTTGAATTTAGCAGTTACTCTTGTAGC |
| ASAP3 | Sence | CACCGCTGTCCACAGGCTTCCTAAATTCAAGAGATTTAGGAAGCCTGTGGACAGCTTTTTTG |
|  | Antisence | GATCCAAAAAAGCTGTCCACAGGCTTCCTAAATCTCTTGAATTTAGGAAGCCTGTGGACAGC |
